# Chromosomal Abnormalities in Miscarriages and Maternal Age: New Insights from the Study of 7118 Cases

**DOI:** 10.3390/cells14010008

**Published:** 2024-12-26

**Authors:** Anna A. Pendina, Mikhail I. Krapivin, Olga G. Chiryaeva, Lubov’ I. Petrova, Elizaveta P. Pashkova, Arina V. Golubeva, Andrei V. Tikhonov, Alla S. Koltsova, Ekaterina D. Trusova, Dmitrii A. Staroverov, Andrey S. Glotov, Olesya N. Bespalova, Olga A. Efimova

**Affiliations:** D.O. Ott Research Institute of Obstetrics, Gynecology and Reproductology, Mendeleevskaya Line 3, 199034 Saint Petersburg, Russia

**Keywords:** miscarriage, chromosomal abnormalities, maternal age, aneuploidy, polyploidy, trisomy, monosomy, karyotype, natural conception, first-trimester pregnancy loss

## Abstract

Chromosomal abnormalities of the embryo are the most common cause of first-trimester pregnancy loss. In this single-center study, we assessed the frequency and the spectrum of chromosomal abnormalities in miscarriages for each year of maternal age from 23 to 44. Cytogenetic data were obtained by conventional karyotyping of 7118 miscarriages in women with naturally conceived pregnancies. Chromosomal abnormalities were identified in 67.25% of miscarriages. The total incidence of chromosomal abnormalities increased with maternal aging; however, its average change for a one-year increase in maternal age differed between age spans, equaling 0.704% in the span from 23 to 37 years and 2.095% in the span from 38 to 44 years. At the age of 38 years, the incidence rate surged sharply by 14.79% up to 79.01% and then increased progressively up to 94% in 44-year-old women. The spectrum of chromosomal abnormalities in miscarriages was the same for each year of maternal age from 23 to 44 years. However, the proportions of particular chromosomal abnormalities differed between karyotypically abnormal miscarriages in younger and older women. The proportions of trisomy 16, polyploidy, monosomy X, mosaic aneuploidies, and structural rearrangements decreased with increasing maternal age. In contrast, the proportions of multiple aneuploidies and regular trisomies 13, 15, 18, 21, and 22 showed an upward trend with maternal aging. To summarize, despite the increase in the total incidence of chromosomal abnormalities in miscarriages with maternal aging, the rate of change differs for younger and older women, being three times lower in the former than in the latter. Moreover, the proportion of some abnormalities in karyotypically abnormal miscarriages shows a steady growth, whereas the proportion of others becomes increasingly low with maternal aging, most probably due to the age-dependent prevalence of different molecular and cellular defects.

## 1. Introduction

Pregnancy loss is a critical medical and social challenge that has been growing in scale over the last several years. Although the outcome is associated with a broad variety of underlying factors, chromosomal abnormalities in the embryo are among the most widely spread causes. Publications by fellow investigators report that the incidence rate of karyotype abnormalities responsible for first-trimester miscarriage varies from 40% to 76% [1,2,3,4,5,6,7,8,9]. The spectrum of chromosomal abnormalities in miscarriages is much broader than that in the postnatal period of human development. Miscarriages are characterized by polyploidies, aneuploidies, structural chromosomal rearrangements, and mosaic abnormalities. Available research data suggest that the incidence rate of certain chromosomal abnormalities in miscarriages varies, which is apparently conditioned by the size of studied samples and their characteristics, such as maternal age, method of conception, gestational age at miscarriage, and others [10,11,12,13,14,15].

Currently, maternal age is the only accepted and unarguable factor associated with an elevated rate of karyotypically abnormal conceptions [10,12,16,17,18,19]. The link between an abnormal embryonic karyotype and maternal age was discovered in the 1930s, when evidence was obtained showing that the risk of Down syndrome in a newborn is augmented with maternal age [20]. The major cause behind this link is that with the mother’s age, numerous mechanisms involved in meiotic chromosome segregation in human oocytes can run out of control. Meiosis errors result in chromosome nondisjunction and the formation of genetically unbalanced gametes [21,22,23,24,25,26]. Once fertilized, such gametes produce aneuploid embryos. Miscarriages are characterized by a significant variation in the incidence rates of different aneuploidies, largely due to the specific size and structure of each chromosome, as well as maternal age [17,27,28,29,30,31,32]. This fact evidences that aneuploidy development is induced by a myriad of defected processes, rather than a single unique error. Meiotic cell division errors can be followed by fertilization abnormalities and chromosome segregation errors during mitotic divisions at the preimplantation stage. Such combinations may result, for example, in hypo- or hypertriploidy when aneuploid gamete fertilization coincides with fertilization errors. Furthermore, an embryo can develop various chromosomal defects as a result of numerous factors, including endogenous (e.g., meiotic defects or oocyte immaturity) and exogenous (e.g., dysfunctional oocyte microenvironment, hormonal imbalances, etc.) impacts. Despite the solid body of evidence showing that both types of impacts are closely interlinked with maternal age, chromosomal abnormalities leading to pregnancy loss are nevertheless typical—though to a different measure—in women of any reproductive age [13,15,30,33].

It has not been elucidated so far how the patterns of different chromosomal abnormalities in miscarriages change with maternal age. The main reason for the lack of these data is that the majority of studies are based on a relatively small number of miscarriages, rarely exceeding 1000 cases [6,7,9,11,12]. This limits the reliable statistical analysis of the prevalence of particular chromosomal abnormalities in miscarriages at different maternal ages. Therefore, in the present study enrolling 7118 cases of pregnancy loss, we aimed to assess the frequency and the spectrum of chromosomal abnormalities in miscarriages for each year of maternal age from 23 to 44.

## 2. Materials and Methods

### 2.1. Study Samples

The study retrospectively included 7118 cases of miscarriages, which were karyotyped in D.O. Ott Research Institute of Obstetrics, Gynecology, and Reproductology (St. Petersburg, Russia) in 2007–2024. All of these cases were naturally conceived singleton pregnancies with a developmental arrest in the first trimester—before 12 weeks of gestation. The women’s age ranged from 23 to 44 years (mean 32.58 ± 5.09).

The products of conception were obtained by uterine curettage in hospitals of St. Petersburg, and sent to our center in 0.9% NaCl. For karyotyping, chorionic villi were selected using the Leica M125 stereomicroscope.

### 2.2. Karyotyping of Chorionic Villi

Metaphase slide preparations were made from the chorionic villi by a ‘direct’ technique routinely used in our laboratory [34,35,36]. The ‘direct’ technique yields metaphases by spontaneously dividing cytotrophoblast cells from chorionic villi without their culturing, thus eliminating the probability of contaminating the slide preparations with maternal mitoses. In brief, chorionic villi were incubated in 0.9% sodium citrate containing 2.5 μg/mL of colchicine, fixed with a freshly prepared fixative (ethanol:glacial acetic acid, 3:1) and treated with drops of 60% acetic acid for tissue maceration on the slides. Suspension of chorionic cells was spread on the slides, fixed, and then dried at +55 °C for at least 12 h.

The slide preparations were stained with Hoechst 33258 fluorescent dye and treated with actinomycin D to produce chromosome banding. The conventional cytogenetic analysis was performed at a 400–550-band level on 7–15 QFH/AcD-stained metaphases per each case.

### 2.3. Statistical Analysis

Statistical analysis was performed with GraphPad Prism v. 6.0. The Chi-square test with Yates’ correction was used to compare categorical variables; the null hypothesis was rejected if *p* < 0.05. The correlations were assessed using the nonparametric Spearman test. The beta coefficients were assessed using linear regression analysis.

## 3. Results

### 3.1. Incidence of Abnormal Karyotype in Miscarriages and Maternal Age

A sample of 7118 first-trimester miscarriages in women aged 23 to 44 years (mean 32.58 ± 5.09) with natural conceptions was analyzed. The incidence of chromosomal abnormalities in the total sample was 67.25% (4787 out of 7118 cases).

**Figure 1:**
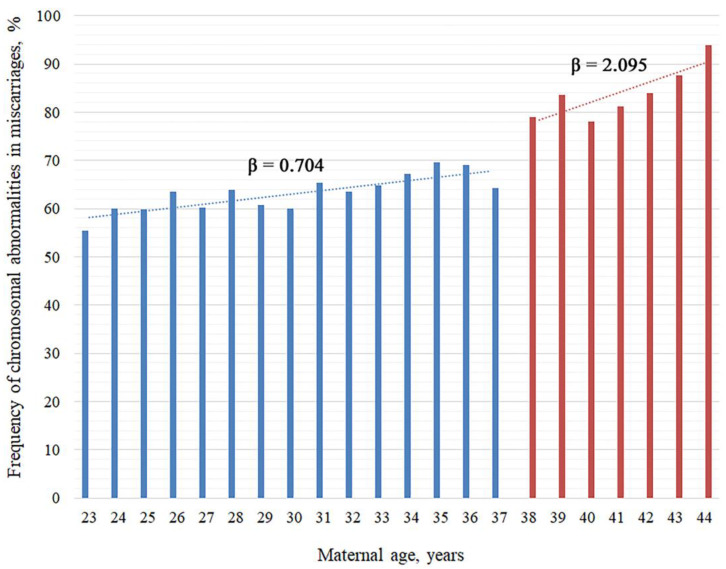
The incidence of chromosomal abnormalities in miscarriages in women aged 23 to 44 years with natural conceptions. The beta coefficients show the average growth in the incidence of abnormal karyotype in miscarriages for a one-year increase in maternal age at the spans of 23–37 years and 38–44 years.

We assessed the incidence of chromosomal abnormalities in miscarriages for each year of maternal age from 23 to 44 (Figure 1). The rate of chromosomal abnormalities in miscarriages varied from 55.38% to 93.85%, with the lowest incidence in 23-year-old women and the highest in 44-year-olds. Thus, the maximum difference in abnormal karyotype frequency reached 38.47% between miscarriages in 23-year-old and 44-year-old women. Notably, women aged 23 to 37 years were characterized by a high chromosomal abnormality rate in miscarriages, equaling or exceeding 60% except for a rate of 55.38% in 23-year-olds. However, no remarkable alterations among ages were observed within this specific age span, with a difference of only 14.12% between the minimal (55.38%) and the maximal (69.50%) frequency of karyotypically abnormal miscarriages. In contrast, women aged 37 to 44 years exhibited a steeper increase in abnormal karyotype rates in miscarriages with age, ranging from 64.23% to 93.85% with a span of 29.62%.

To identify the maternal age prone to the most dramatic surge in the incidence of chromosomal abnormalities leading to miscarriage, we compared the prevalence of karyotypically abnormal miscarriages between each precedent/subsequent age group across the selected age span. The Chi-square test with Yates’ correction showed that the 37/38 age pair was the only one to demonstrate a statistically significant upsurge in the frequency of karyotypically abnormal miscarriage (*p* < 0.0001, Table 1), from 64.22% at the age of 37 years to 79.01% at the age of 38 years. In the subsequent maternal years, a high level of chromosomal abnormality in miscarriages was maintained except for a slight drop to 78.11% at 40 years of age. It should be highlighted that for each year of maternal age, 65 to 514 miscarriage cases were analyzed (Table 1), ensuring a high validity of our results. This allows us to assume that the maternal age of 38 years is associated with a sharp increase in the incidence of chromosomal abnormalities observed in miscarriages after natural conception. Importantly, the calculation of the beta coefficients showed that average growth of incidence rate for a one-year increase in maternal age in women of 38 to 44 years equaled 2.095%, three times higher than that in women of 23 to 37 years (0.704%) (Figure 1).

Thus, despite the increase in the total incidence of chromosomal abnormalities in miscarriages with maternal aging, the rate of change differed between the age spans of 23–37 years and 38–44 years.

### 3.2. The Spectrum of Chromosomal Abnormalities in Miscarriages and Maternal Age

**Figure 2:**
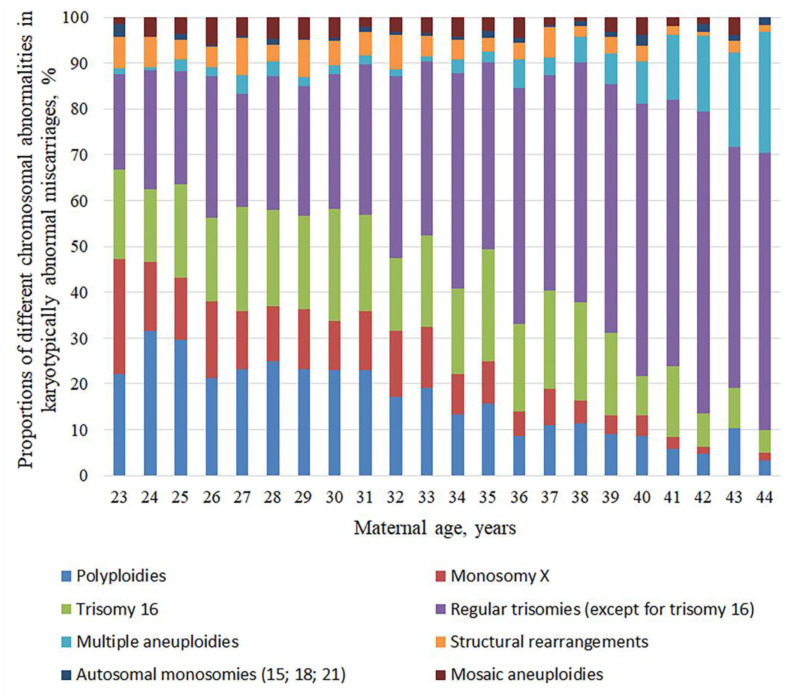
The proportions of different cytogenetic categories in karyotypically abnormal miscarriages in women aged 23 to 44 years with natural conceptions.

The cytogenetic analysis of first-trimester miscarriages showed that poly- and aneuploidies were predominant in the chromosomal abnormality spectrum, while structural rearrangements and mosaicism were less common. The observed chromosomal abnormalities were classified into eight cytogenetic categories based on their causes and frequency: (1) polyploidy, including triploidy, tetraploidy, hypotriploidy and hypertriploidy; (2) monosomy X; (3) regular trisomy 16; (4) regular trisomies of chromosomes 2–15, 17, 18, and 20–22, and disomy X and Y; (5) multiple aneuploidies, including multiple trisomies and trisomies combined with monosomies; (6) structural chromosomal rearrangements; (7) autosomal monosomies 15, 18, and 21; and (8) mosaic aneuploidies (Table 2). To identify maternal-age-associated changes in the spectrum of chromosomal abnormalities, we calculated the proportions of cytogenetic categories in karyotypically abnormal miscarriages for each year of maternal age from 23 to 44 (Figure 2).

Notably, each year of maternal age from 23 to 44 was characterized by miscarriages with chromosomal abnormalities from all of the eight cytogenetic categories, although the proportions varied across the groups depending on maternal age. Polyploidy, monosomy X, trisomy 16, mosaic abnormalities, and structural rearrangements showed an overall proportion decrease with maternal age, whereas regular trisomies and multiple aneuploidies demonstrated an upward trend. Interestingly, in miscarriages in 23-year-old women, the aggregated proportion of polyploidies, monosomy X, trisomy 16, mosaic aneuploidies, and structural rearrangements accounted for ~75% of the total chromosomal abnormalities, dropping down to as low as 11% by the age of 44 years. In contrast, the aggregated proportion grew with maternal age for regular trisomies in all chromosomes, except chromosome 16, and for multiple trisomies (from ~25% in 23-year-old women to ~89% in 44-year-olds). In addition, regular trisomies demonstrated a smooth pattern of incidence rate increase throughout the maternal age span, while multiple aneuploidies showed a steep surge in women aged 36 or older. Moreover, a remarkable maternal-age-dependent trend was observed for trisomy 16 in karyotypically abnormal miscarriages: its proportion decreased from 19.44% in 23-year-old women to 4.92% in 44-year-olds. A similar decline with maternal age, though not such a dramatic one, was observed for mosaic aneuploidies, structural rearrangements, and autosomal monosomies.

Thus, in contrast to the spectrum of chromosomal abnormalities in miscarriages, which remained unchanged with maternal age, the proportions of several cytogenetic categories were susceptible to pronounced and contrasting maternal-age-dependent alterations.

### 3.3. Maternal-Age-Associated Changes Across Proportions of Chromosomal Abnormalities in Karyotypically Abnormal Miscarriages

In the next step, we analyzed the strength and the direction of the correlation between maternal age and the incidence rate of individual chromosomal abnormalities in karyotypically abnormal miscarriages: the most common trisomies, 13, 15, 16, 18, 21, and 22, multiple aneuploidies, monosomy X, polyploidies, mosaic aneuploidies, and structural chromosomal rearrangements (Table 2). For every chromosomal abnormality, the nonparametric Spearman test showed a significant correlation between the incidence rate and maternal age; the direction—positive or negative—varied across abnormalities.

**Figure 3:**
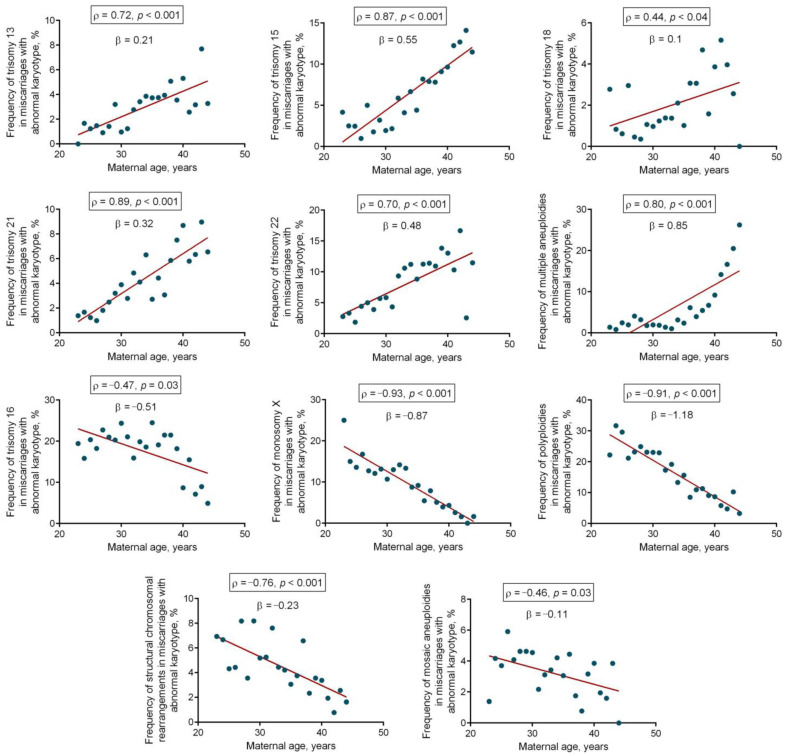
Correlations between the maternal age and the incidence of different chromosomal abnormalities in miscarriages with an abnormal karyotype. Statistically significant correlations are framed (*p* < 0.05, the nonparametric Spearman test). The beta coefficients show the average change in the incidence of chromosomal abnormality for a one-year increase in maternal age.

The strongest positive correlation was detected between maternal age and the incidence of miscarriages marked by trisomies 21 and 15 (ρ = 0.89, *p* < 0.0001 and ρ = 0.87, *p* < 0.0001, respectively), as well as multiple aneuploidies (ρ = 0.8, *p* < 0.0001). The incidence of trisomy 13 and 22 in miscarriages showed a less remarkable elevation with maternal aging (ρ = 0.72, *p* < 0.0001 and ρ = 0.70, *p* < 0.0001, respectively), whereas the incidence rate for trisomy 18 was characterized by a moderate positive correlation with maternal age (ρ = 0.44, *p* = 0.04) (Figure 3). In stark contrast, monosomy X and polyploidy rate in miscarriages decreased markedly with increasing maternal age (ρ = −0.93, *p* < 0.0001 and ρ = −0.91, *p* < 0.0001, respectively), while structural rearrangements (ρ = −0.76, *p* < 0.0001), trisomies 16 (ρ = −0.47, *p* = 0.02), and mosaic aneuploidies (ρ = −0.46, *p* = 0.03) diminished less remarkably (Figure 3).

The linear regression models showed that among chromosomal abnormalities increasing in frequency with maternal aging, multiple trisomies, trisomy 15, trisomy 22, and trisomy 21, were characterized by the most pronounced average annual growth: 0.85%, 0.55%, 0.48%, and 0.32%, respectively. The average growth in the incidence of trisomy 13 and trisomy 18 for a one-year increase in maternal age was lower: 0.21% and 0.1%, respectively. Among chromosomal abnormalities decreasing in proportion with maternal aging, polyploidy (−1.18%), monosomy X (−0.87%), and trisomy 16 (−0.51%) demonstrated the most prominent annual decline. A less prominent annual decline was typical for the incidence of structural chromosomal rearrangement (−0.23%) and mosaic aneuploidies (−0.11%) (Figure 3).

Therefore, the incidence rate of particular chromosomal abnormalities in karyotypically abnormal miscarriages is strongly associated with maternal age, showing a direct or inverse relationship depending on the abnormality type. Polyploidy, monosomy X, trisomy 16, structural rearrangements, and mosaic aneuploidies in miscarriages are mostly associated with younger maternal age, whereas trisomies 13, 15, 18, 21, and 22 and multiple aneuploidies affect miscarriages in older women.

## 4. Discussion

Chromosomal abnormalities in the embryo are the most common cause of miscarriage. Such abnormalities arise due to the following major factors: (1) participation of aneuploid gametes in fertilization, (2) fertilization errors, or (3) chromosome segregation error during mitoses, in particular during cleavage divisions [31,32,37]. The formation of aneuploid gametes is most characteristic of oogenesis and is caused by disruptions in multiple mechanisms involved in meiotic divisions: meiotic recombination [23,38,39,40,41], cohesion [30,42,43], and spindle formation [24,29,30]. A bulk of research provides evidence suggesting that oocyte aneuploidy increases with a woman’s age [16,18,19,30,32,44,45,46,47]. However, the age threshold associated with critically elevated risks for chromosomal abnormality varies across publications from 35 [10,12,48] to 40 years [11,49]. In this study, a significant (almost 15%) surge in the rate of fetal chromosomal abnormality was observed among women at the age of 38 years, followed by an incremental rise to 94% by 44 years. Although not without differences, a similar relationship between aneuploidy rate and maternal age was revealed in the PGT-A results from 15,169 blastocysts [19]. According to Franasiak et al., a 7.1% surge in aneuploidy frequency rate in blastocysts was observed at the age of 37 years, bringing it to 42.6%, which is still 22% less frequent than our research suggests [19]. The latter could be explained by the difference between natural and ART-assisted conception, presuming a lower incidence of chromosomal abnormalities in ART-assisted than in naturally conceived arrested pregnancies [14,34,50,51]. The underlying broad range of causes may include in vitro selection of aneuploid embryos, especially at cleavage stages [14,51]. It should also be noted that, unlike the authors of the present study, Franasiak et al. investigated blastocysts which, if transferred into the uterine cavity, may have resulted in pregnancies that would have terminated when the women were 38 years old [19]. This suggests that the age of 38 years is a critical threshold marked by a significant surge in karyotypically abnormal embryos. Nevertheless, under the maternal age of 38 years, the chromosomal abnormality rate in miscarried embryos is also rather high [13,15,30,33]. Our study shows that in women aged 23 to 37 years with naturally conceived pregnancies, miscarriages exhibited chromosomal abnormalities of mean 60%, ranging from 55% to 69%. Remarkably, an oscillation of abnormal karyotype incidence was observed in miscarriages among women aged 23 to 37 years, with the average annual growth of 0.704%, which contrasted with a sharp surge in women aged 38 followed by a gradual rapid increase in the abnormal karyotype rate: 2.095% per each year of maternal age, on average.

For the proportions of particular chromosomal abnormalities in miscarriages, however, the association with maternal age differs from the overall incidence rate. Our study outlined eight cytogenetic categories showing a direct or inverse correlation with maternal age. Karyotypically abnormal miscarriages in younger women were characterized by the prevalence of such abnormalities as polyploidy, monosomy X, trisomy 16, mosaic aneuploidies, and structural rearrangements—while regular (except trisomy 16) and multiple trisomies prevailed among miscarriages with abnormal karyotype in women of advanced maternal age. Although all of the abnormalities were identified in miscarriages in women of every year of age from 23 to 44, the share of each chromosomal abnormality in the overall spectrum of karyotype pathology varied across age groups. With age, the incidence rate of polyploidy, monosomy X, and trisomy 16 drops significantly, while multiple trisomies and regular trisomy 15 and 21 show an upward trend. Such age-dependent shifts are likely explained by the fact that women of younger and older reproductive age are affected by disruptions in different molecular and cellular mechanisms. Polyploidy, particularly triploidy, commonly originates from fertilization errors and shows a higher incidence in younger women. According to Jacobs et al., triploidy is predominantly of diandric origin, resulting from dispermic fertilization in 66.4% of cases and produced by diploid sperm in 23.6%, while digynic triploidy originating from fertilized diploid oocyte accounts for only 10% [52]. Other publications provide evidence suggesting an incidence rate of 69% [53] to 77% [54] for diandric triploidy and 17% [54] to 31% [53] for digynic triploidy. A matter to consider is that dispermic fertilization can be attributed to a broad range of factors, e.g., type of ovarian stimulation [55], hormonal changes [56,57,58], immature oocyte [59,60,61], and a high count of hypermotile and [58,59,62] morphologically normal spermatozoa [58]. Evidence suggests that immature oocytes have a remarkably higher susceptibility to polysperm fertilization than mature oocytes: 32% vs. 6%, respectively [59]. Immature oocytes may fail to block polyspermy due to the lack of cortical granules and eventual abnormal cortical reaction [59]. Younger women were reported to produce immature oocytes with minor granularity [63], which can inhibit polyspermy blocks and lead to fertilization errors. Therefore, a higher incidence of polyploidy in miscarriages in younger women is most probably associated with the prevalence of immature oocytes conducive to fertilization errors.

In contrast, aneuploidy is driven by a different type of defects—meiotic errors including meiosis I nondisjunction (MI NDJ) [21], precocious separation of sister chromatids (PSSC) [22], and reverse segregation (RS) [25]. Grunh et al. observed the prevalence of various meiotic errors throughout oogenesis in teenage girls, young women, and the advanced-maternal-age group [30]. MI NDJ is more common in younger women, whereas PSSC and RS are generally more frequent in the middle and advanced reproductive age. Although affecting all age groups, the three types of errors contribute differently to the aggregate incidence of meiotic errors, following a U-curve according to the woman’s age [30,31]. The type of meiotic error is strongly associated with chromosomal size and structure [27]. For example, aneuploidies involving large metacentric and submetacentric chromosomes usually derive from the MI NDJ [30,31], more common in young women. Aneuploidies involving acrocentric chromosomes mostly derive from PSSC or RS, which are more common in women of advanced maternal age [17,28,29,30]. The abovementioned facts may underlie the prevalence of miscarriages with monosomy X—large and submetacentric chromosome—in younger women enrolled in our study, while abnormal conceptions in women of advanced maternal age are often characterized by trisomies of acrocentric chromosomes 15, 21, and 22.

The intriguing result of the present study is an observed inverse correlation between maternal age and the incidence rate of trisomy 16 in miscarriages, standing at ~20% of the total abnormalities in 23-year-old women and ~5% in 44-year-olds. Fellow investigators argue that in the overwhelming number of cases, trisomy 16 evolves during oogenesis due to the MI NDJ [23,64,65]—a meiotic error typical of younger women [30]. This may be explained by a specific structure of chromosome 16, which represents a submetacentric with a large pericentromeric heterochromatin region reaching up to half of the long arm. Large pericentromeric heterochromatin may contribute to excessive sister chromatid cohesion, and therefore, retention of homologous chromosomes within bivalent pairs. The latter may impede the segregation of bivalents at the anaphase I, most probably being a major cause of homologous chromosome nondisjunction in meiosis I during oogenesis. It is therefore critical to consider the close link between the chromosome size and structure, on the one hand, and the pattern of homologous recombination responsible for successful segregation, on the other hand. Though fundamental, homologous recombination abnormalities are presumably not the only aneuploidy-inducing factor. Aneuploidies can be incurred by other errors, such as disrupted sister chromatid cohesion [66,67], kinetochore formation and morphology defects [28], and errors of spindle assembly mechanics [68] and cell cycle control [69]. The overall evidence shows that aneuploidy is induced by a multitude of factors, rather than a single cause. Moreover, the observed differences in the incidence rate of chromosomal abnormality in miscarriages suggest that depending on morphology, the susceptibility of chromosomes to a particular range of aberrations may vary.

The limitations of the present study should also be mentioned. Firstly, this is a single-center study performed in a small geographic area; therefore, potential biases in the patients’ demographics are not excluded. Secondly, despite karyotyping being a “gold standard” for chromosome study, microstructural chromosomal abnormalities can be overlooked in the analysis.

In conclusion, it should be noted that large sample sizes are a fundamental prerequisite in any medical research. Our study analyzed 7118 miscarriages from women aged 23 to 44 years. Such an extensive sample is deemed substantial for this type of study, allowing us to obtain novel and reliable results concerning the incidence rate and the spectrum of chromosomal abnormalities in miscarriages assessed for each year of maternal age within a 22-year age span (in women aged 23 to 44 years). In women aged 23 to 37 years, the overall incidence of abnormal karyotype in miscarriages remained at a substantially high level, with a mild increase followed by a sharp surge at the age of 38 years and stepwise rapid increase in the following years. In stark contrast to the total frequency assessment, the incidence rate of specific chromosomal abnormalities in karyotypically abnormal miscarriages revealed vastly diversified dynamics, with some abnormalities becoming progressively common and others increasingly rare with maternal aging. Such a divergent effect of maternal age on the proportions of particular chromosomal pathology may stem from different molecular and cellular mechanisms behind specific abnormalities. The obtained results highlight the clinical relevance of karyotyping the miscarriages of patients of any age, including young women. Profound knowledge of particular chromosomal abnormalities is required for efficient prevention of miscarriage, as well as medical and genetic counselling for couples who have experienced pregnancy loss. Evidence regarding the type of chromosomal abnormality can pave the way towards outlining critical screening procedures for patients or couples, as well as developing efficient pregnancy planning strategies.

## Figures and Tables

**Table 1:** Data on normal and abnormal karyotype cases in miscarriages in women of 23 to 44 years with natural conceptions.

Maternal Age, Years	Miscarriages with Abnormal Karyotype, *n*	Miscarriages with Normal Karyotype, *n*	Total Number of Miscarriages, *n*	Frequency of Abnormal Karyotype in Miscarriages, %	*p*-Value (Chi-Square with Yates’ Correction)
23	72	58	130	55.38	-
24	120	80	200	60.00	0.47
25	162	109	271	59.78	0.96
26	203	117	320	63.44	0.41
27	220	146	366	60.11	0.41
28	281	159	440	63.86	0.31
29	281	182	463	60.69	0.36
30	308	206	514	59.92	0.86
31	323	171	494	65.38	0.08
32	289	166	455	63.52	0.59
33	292	159	451	64.75	0.75
34	285	139	424	67.22	0.48
35	294	129	423	69.50	0.52
36	293	132	425	68.94	0.92
37	228	127	355	64.23	0.19
**38**	**256**	**68**	**324**	**79.01**	**<0.0001**
39	253	50	303	83.50	0.18
40	207	58	265	78.11	0.13
41	155	36	191	81.15	0.50
42	126	24	150	84.00	0.59
43	78	11	89	87.64	0.56
44	61	4	65	93.85	0.31

The frequency of abnormal karyotype in miscarriages increases significantly in women aged 38 years compared to 37-year-old women (*p* < 0.0001, Chi-square test with Yates’ correction).

**Table 2:** Chromosomal abnormality cases in miscarriages in women of 23 to 44 years with natural conceptions.

Maternal Age, Years	Chromosomal Abnormalities in Miscarriages, Number of Cases													
	Trisomy 13	Trisomy 15	Trisomy 16	Trisomy 18	Trisomy 21	Trisomy 22	Regular Trisomies (2–12; 14; 17; 20), Disomy X and Y	Multiple Aneuploidies	Monosomy X	Polyploidies	Structural Rearrangements	Autosomal Monosomies (15; 18; 21)	Mosaic Aneuploidies	Total
23	0	3	14	2	1	2	7	1	18	16	5	2	1	72
24	2	3	19	1	2	4	19	1	18	38	8	0	5	120
25	2	4	33	1	2	3	28	4	22	48	7	2	6	162
26	3	2	37	6	2	9	41	4	34	43	9	1	12	203
27	2	11	50	1	4	11	25	9	28	51	18	1	9	220
28	4	5	59	1	7	11	54	9	34	70	10	4	13	281
29	9	9	57	3	9	16	34	5	37	65	23	1	13	281
30	3	6	75	3	12	18	49	6	33	71	16	2	14	308
31	4	7	68	4	9	14	68	6	42	74	17	3	7	323
32	8	17	46	4	14	27	45	4	41	50	22	2	9	289
33	10	12	58	4	12	31	42	3	39	56	13	2	10	292
34	11	19	53	6	18	32	48	9	25	38	12	2	12	285
35	11	13	72	3	8	26	59	7	27	46	9	4	9	294
36	11	24	56	9	13	33	61	18	16	25	11	3	13	293
37	9	18	49	7	7	26	40	9	18	25	15	1	4	228
38	13	20	55	12	15	28	46	14	13	29	6	3	2	256
39	9	23	46	4	19	35	47	17	10	23	9	3	8	253
40	11	20	18	8	18	27	39	19	9	18	7	5	8	207
41	4	19	24	8	9	16	34	22	4	9	3	0	3	155
42	4	16	9	5	8	21	29	21	2	6	1	2	2	126
43	6	11	7	2	7	2	13	16	0	8	2	1	3	78
44	2	7	3	0	4	7	17	16	1	2	1	1	0	61

## Data Availability

Data may be obtained by contacting the corresponding author.
